# Lost in transition? Perceptions of health care among young people with mental health problems in Germany: a qualitative study

**DOI:** 10.1186/s13034-018-0249-9

**Published:** 2018-08-07

**Authors:** Sabine Loos, Naina Walia, Thomas Becker, Bernd Puschner

**Affiliations:** Section Process-Outcome Research, Department of Psychiatry II, Ulm University, Ludwig-Heilmeyer-Str. 2, 89312 Günzburg, Germany

**Keywords:** Mental health service, Youth mental health, Transition, Qualitative research, Health care needs

## Abstract

**Background:**

The transitioning of young patients from child and adolescent to adult mental health services when indicated often results in the interruption or termination of service. The personal views of young service users on current clinical practice are a valuable contribution that can help to identify service gaps. The purpose of this qualitative study was to explore the perceptions of health care of young people with mental health problems in the transition age range (16–25 years), and to better understand health behaviour, care needs and the reasons for disengaging from care at this point in time.

**Methods:**

Seven group discussions and three interviews were conducted with 29 young people in this age range. Discussions were audio-taped, transcribed verbatim and analysed following the reconstructive approach of R. Bohnsack’s documentary method.

**Results:**

An overarching theme and nine subthemes emerged. Participants displayed a pessimistic and disillusioned general attitude towards professional mental health services. The discussions highlighted an overall concern of a lack of compassion and warmth in care. When they come into contact with the system they often experience a high degree of dependency which contradicts their pursuit of autonomy and self-determination in their current life stage. In the discussions, participants referred to a number of unmet needs regarding care provision and strongly emphasised relationship issues. As a response to their care needs not being met, they described their own health behaviour as predominantly passive, with both an internal and external withdrawal from the system.

**Conclusions:**

Research and clinical practice should focus more on developing needs-oriented and autonomy-supporting care practice. This should include both a shift in staff training towards a focus on communicative skills, and the development of skills training for young patients to strengthen competences in health literacy.

**Electronic supplementary material:**

The online version of this article (10.1186/s13034-018-0249-9) contains supplementary material, which is available to authorized users.

## Background

In recent years, young people aged 16–25 with mental health problems have received increasing attention in research and clinical practice as a vulnerable group with special health care needs. Prevalence rates for mental disorders in this life period are high. For young people in the US, it was found out that 18.7% aged 18–25 years had some form of mental illness, of which 3.9% had a serious mental illness [1]. Similarly, in Germany, the point prevalence of mental illness in 14- to 17-year-olds is 17.8%, with a significant increase of emotional problems over time [2]. The onset age for most persistent mental disorders falls between 12 and 24 years [3]. At the same time, compared to all age groups across the life span, this age group has the lowest rate of access to mental health care [4].

One major reason for the lack of continuity of care is the complexity and the fragmented organisation of mental health services for young people worldwide [5, 6]. In Germany, the official age for transitioning to adult care is 18 years, with various exceptions in clinical practice. There is a wide range of special mental health services financed by health insurance companies, community organisations or federal states (e.g. in- and outpatient psychiatric or psychosomatic clinics, outpatient psychiatric clinics or psychotherapies, outreach clinics). A lack of knowledge of where to go for which problems, as well as fewer offers of the low-threshold and less formal services usually preferred by young patients, make it difficult to find the right place to go [7].

Furthermore, differences in care philosophy and working practice between child and adolescent and adult mental health services (CAMHS, AMHS) may hinder effective collaboration [8, 9]. Often clinicians fail to actively refer patients from CAMHS to AMHS, or young people refuse such a referral [10]. Effective programmes to smooth the transition are rare and face logistical and organisational barriers [11, 12]. Personal obstacles for young people receiving adequate mental health care include self-stigma, not recognising symptoms as warning signs, a preference for self-reliant actions, and a lack of mental health literacy [6, 13, 14]. An empathetic, effective, and meaningful practice in mental health service can support youth in building resilience [15].

Qualitative studies provide insight into the subjective experiences of young people and can identify factors which contribute to a successful transition, such as: (i) feeling connected and supported in their relationships to significant others [16–18]; (ii) helpful and reliable connections with health care professionals who are attentive and respectful towards their young patients and who motivate them to continue care [19–21]; (iii) realistic expectations of the care provision provided by AMHS [21]. Moreover, experiences of stigmatising attitudes towards mental illness are a common theme among transitioning youth, which often results in social withdrawal [16, 19]. Practical suggestions for improving and tailoring care from patients’ and carers’ points of view include a gradual approach to treatment, transfer planning meetings, peer involvement, and elements of social support [22, 23].

In summary, there are some hints for needs-adapted professional care for young people with mental health problems. In addition, young people’s implicit motives for discontinuing health service use, their care needs and the determinants of health behaviour could extend our knowledge of how to adapt service culture. To date, group discussions as a qualitative research method have hardly been tested [18], but they are an appropriate method for investigating the views and experiences of youth who share a common social context, and for considering the interactive group process. By using a reconstructive qualitative approach, this study explores the personal experiences of patients aged 16–25 years with service utilisation. The study’s research questions are the following:

(1) What are young patients’ perceptions and evaluations of health care during the transition from CAMHS to AMHS? (2) What are their (mental) health care needs? And (3) Which factors influence their health behaviour?

## Methods

The study followed a qualitative-explorative and reconstructive approach to gain insight into the action-guiding, common and tacit forms of knowledge of groups on the basis of anecdotes or beliefs [24]. Group discussions are especially appropriate for exploring milieu-specific structures and collective experiences, and are an established method for exploring the attitudes and views of young people [18, 22, 25]. During the research process, however, we were confronted with three potential study candidates who refused to take part in a group discussion for personal reasons. Since they were seen as providing a significant contribution to the study aims and in order to fully explore the field, we decided to give them the opportunity to be personally interviewed. The present paper followed the consolidated criteria for reporting qualitative research (COREQ) checklist [26].

### Research team and reflexivity

The research team consisted of three female researchers (SL, NW, and a student assistant, IT, see “Acknowledgements”) who were all trained and experienced in conducting qualitative research and group discussions. The research team was not involved in patient care and did not know the participants prior to study inclusion. The researchers explained their personal positions to the team and their interest in the research topic to the participants.

### Sampling method

**Fig. 1:**
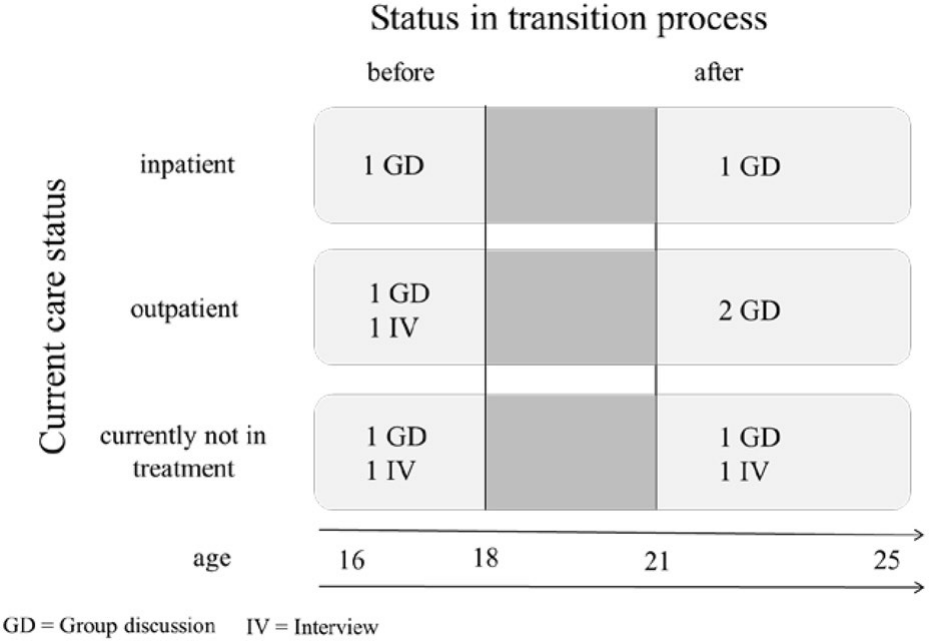
Distribution of groups and interviews according to the dimensions of the sampling plan

We developed a sampling plan prior to recruitment. Relevant parameters were the current status of participants in the process of transition from CAMHS to AMHS (before or after) and current treatment status (inpatient, outpatient, or currently not in treatment, see Fig. 1). The rationale for case selection was to obtain a maximum level of variation to enhance the external validity of findings. Data saturation was continuously discussed. We adhered to the sampling strategy as much as possible and observed both general and continually emerging themes over the course of the group discussions.

### Development of an interview guide

A flexible interview guide with open-ended questions as prompts for discussion was developed in an iterative process by the research team. The opening stimulus question was “Tell us about your personal experiences of care for your mental health problems”. During discussion, topics (and examples of questions and prompts) were provided. A flexible application of the guide allowed the moderator to individually adapt to the specific dynamics of each individual group discussion. The ideal course of a discussion was intended as follows: [1] warming up [2], main interview phase including queries by the moderator to explore issues raised by participants [3], phase of introducing relevant research topics not yet discussed by the group [4], confrontation phase, where contradictions, impressions or interpretations were addressed by the moderator, and [5] conclusion phase. The interview guide was adapted after pilot-testing in a test group discussion.

### Participants and recruitment

Recruitment took place between September 2015 and July 2016 at four study sites in Germany: at the in- and outpatient services of two of Ulm University’s Departments of Psychiatry and Psychotherapy, one for children and adolescents (Child and Adolescent Psychiatry and Psychotherapy, Ulm) and the other for adults (Department of Psychiatry II, Clinic for Psychiatry, Psychotherapy and Psychosomatics at the Bezirkskrankenhaus Günzburg); at an outpatient outreach clinic for families, children and adolescents (Psychologische Beratungsstelle Ulm); and at a community mental health clinic for children and adolescents in central Germany (Vitos Klinik Rehberg, Herborn).

Participants were approached via gate-keepers, flyers, and snowballing and gave informed written consent after detailed study information had been provided. If participants were under 18 years old (the age of consent in Germany), informed consent was obtained from both participants and caregivers. All participants received a voucher of 40 €. The study was approved by Ulm University’s Ethics Committee.

Participants were considered eligible if they met the following criteria: [1] aged 16–25 years [2]; at least one personal contact with mental health services prior to the study, either completed or still in progress. Patients with insufficient command of the German language were excluded. After each session, socio-demographic data were obtained using a questionnaire.

### Data collection

Group discussions and interviews took place either in a neutral setting at our research division or in settings familiar to the person or group (e.g. inpatient clinic). In case it was not possible to organise a familiar place for the discussion/interview, we invited the participants to our research division. In some groups the study participants knew each other, while in others they did not know each other. The groups were led by a moderator (NW) and an observer (either IT or SL) who took field notes (e.g. on the seating plan, atmosphere, and non-verbal communication). The moderator’s tasks during the discussion were to listen to the discussion, to create an information-eliciting atmosphere, and to recognise any common concerns and shared experiences within the group. If verbal communication among participants stopped, the moderator encouraged further discussions by repeating or reframing a question or referring to the interview guide.

### Data analysis

The group discussions and interviews were audio-taped and transcribed verbatim. Anonymous code names were given to each participant. Participants were invited to inspect the transcript. Data analysis was based on the reconstructive approach of Bohnsacks’ documentary method [27]. A multi-level approach was undertaken by the coders: [1] transcripts were read independently by SL, NW and IT, and the content of each transcript was structured into paragraphs labelled with open, descriptive headings for the themes and sub-themes which were perceived as meaningful [2]. In a mutually consensual process, relevant paragraphs were selected for further analysis due to their interactive intensity and thematic relevance [3]. For each selected paragraph, an independent discourse analysis was conducted to paraphrase and structure the course of discussion [4]. The constant comparison technique was used to compare the meanings of paragraphs within and between groups and interviews to extract group consensus. Alternative interpretations, and the overall impressions and theorisations of the researchers were taken into account [5]. The themes and categories consented to were transferred to an overall coding tree which was validated and extended repeatedly. There were regular validation sessions held with an external, multidisciplinary interpretation group to independently discuss preliminary results in light of various professional perspectives. Citations of participant statements were translated into English by SL and approved by the co-authors [28].

## Results

Seven group discussions (each with 3–5 participants) and three interviews were conducted, lasting between 59 and 117 min each. A total of 29 participants took part in the study. On average, participants were 20 years old; two-thirds of them were female. Participants’ educational background was mixed and their mental health conditions varied (Table 1).

**Table 1:** Sample description (N = 29)

Characteristics	Values
Age, years; M (SD)	20.3 (3.3)
Sex, female; N (%)	19 (65.5)
Educational degree^a^	
Low track^b^; N (%)	10 (34.5)
Middle track^c^; N (%)	7 (24.1)
High track^d^; N (%)	10 (34.4)
No degree; N (%)	1 (3.4)
Duration of illness^e^	
< 2 years; N (%)	0
> 2 years; N (%)	26 (89.7)
Mental health problem (self-report)^f^	
Schizophrenia spectrum; N (%)	3 (10.3)
Affective spectrum; N (%)	9 (31.0)
Anxiety spectrum; N (%)	3 (10.3)
Eating spectrum; N (%)	2 (6.9)
Personality spectrum; N (%)	4 (13.8)
Attention deficit/hyperactivity spectrum; N (%)	3 (10.3)
Unknown; N (%)	3 (10.3)
Current treatment status	
Inpatient; N (%)	10 (34.5)
Outpatient; N (%)	10 (34.5)
Other treatment; N (%)	6 (20.7)
No treatment; N (%)	3 (10.3)

^a^Still at school or completed; missing = 1

^b^Hauptschule

^c^Realschule

^d^(Fach-)Abitur

^e^Missing = 3

^f^Missing = 2

**Fig. 2:**
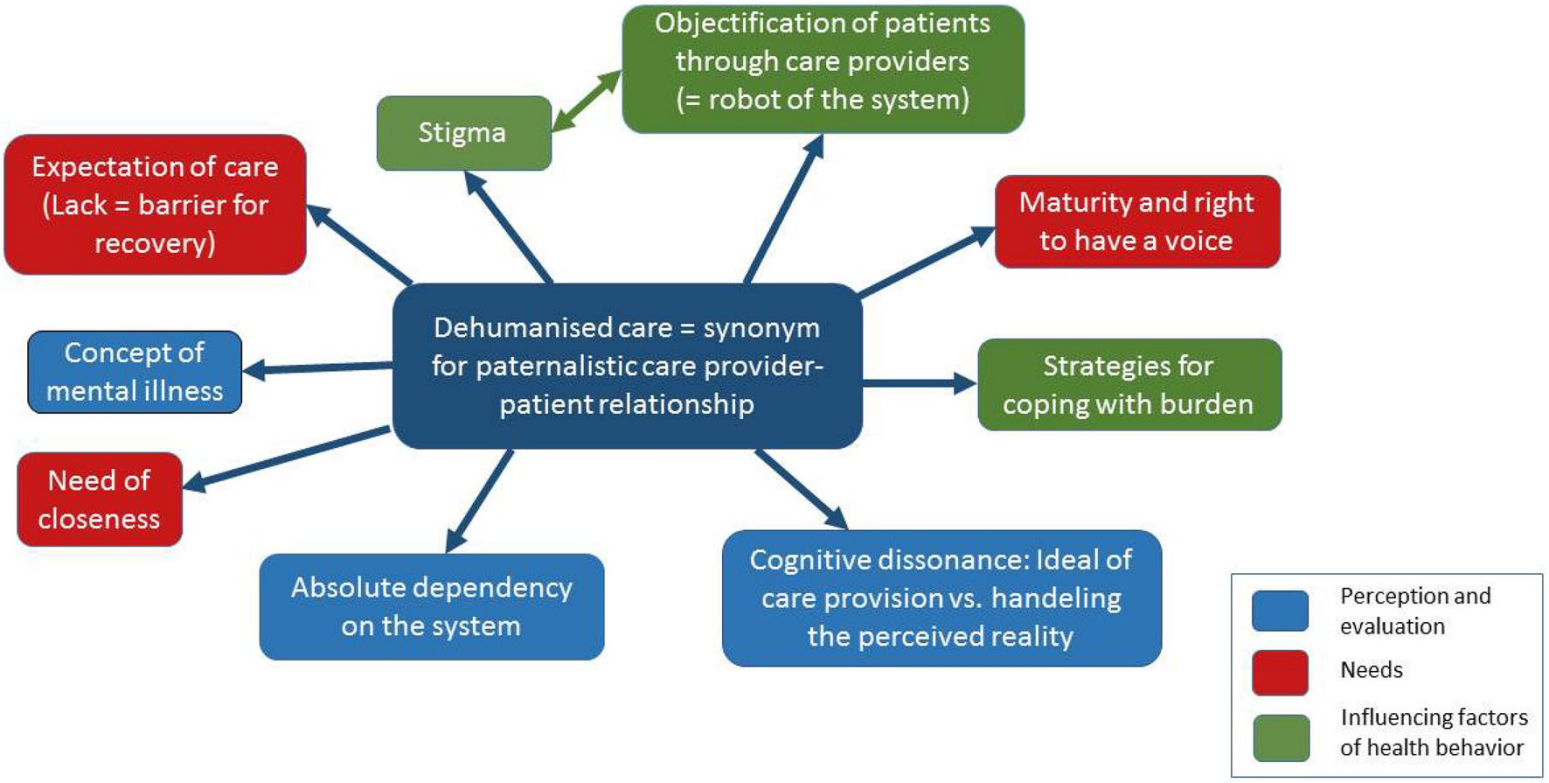
Coding tree as mind map

Overall, 43 descriptive and conceptual codes were extracted during analysing process resulting in a hierarchical structure with four levels. Figure 2 shows part of the coding tree as a mind map, a graphical solution including the codes at level 1 and 2. Described codes are grouped in terms of colour according to the three research questions of the study (for the full coding tree, see Additional file 1: Figure S1).

An overarching main theme emerged at level 1 (“dehumanised care”), and nine subthemes were identified at level 2 which are presented in Table 2 as they relate to the study’s research questions. The main theme and subthemes are described below.

**Table 2:** Summary of research topics and corresponding subthemes at level 2

Research questions	Subthemes (level 2)
Perception and evaluation of care	Absolute dependency on the system Cognitive dissonance (ideal of care vs. reality) Concept of mental illness
Health care needs	Expectations of care Need of closeness Maturity and the right to have a voice
Influencing factors of health behaviour	Stigma Objectification of patients through care providers Strategies for coping with burden

### Main theme: dehumanised care

The overall theme identified was “dehumanised care”. Young people in the study described a lack of humanity, attentiveness and empathy in the care system, often illustrated by descriptions of personal encounters with care providers which they experienced as paternalistic and authoritarian. They felt that their personal needs and concerns were not addressed. This impression on the part of participants seems to be the main factor shaping their predominantly pessimistic view of the care system. They often felt misunderstood and that their needs especially in crisis situations went unnoticed. Participants often regarded staff as indifferent, obedient to the system, not interested in individual cases, and in thrall to stereotypical thinking where they label and classify patients according to preconceived diagnostic categories. Participants experience diagnosis as an arbitrary act unrelated to the individual and their situation.

*Because I still have another life. I also have a life as a human being and not just as a sick person (female, group discussion (GD): inpatient care after the transition process).*

*One just realises that the ward round is led by people who are not really interested in their work. I do not have the impression that anybody is seriously interested. They are all so apathetic… (female, GD: inpatient care after the transition process).*

*The doctors quickly get a certain impression of someone. They listen to us but they quickly say (…) you have a little bit of this and much more of that. You know, they are also just humans (…) because in my case*—*I was recently diagnosed with schizophrenia*—*I’ve never been schizophrenic before; and this was a mistake made by a doctor who wrote that down incorrectly; and I think ok, I am here because of depression and now I have schizophrenia… (male, GD: outpatient care after the transition process).*

### Research question 1: perception and evaluation of health care

Young people regarded themselves as dependent on the care system, a fact that conflicted with their pursuit of autonomy and self-determination in their current life stage (“absolute dependency on the system”, Fig. 2).

*In general, if you get some psychotropic drugs like from the “nice” lady where I am; I say I would like this and that but she doesn’t give a damn. She does what she wants to because she has an idea; I say ok, this is too much, yes, it will be ok (female, GD: outpatient care after the transition process).*

Participants reported that when they felt in need of care, they wanted to be taken seriously and treated as human beings with emotions. This would be in line with their ideal of care provision but often contrasted with their experiences and perceived reality. This caused a dissonance which they tried to solve. Furthermore, participants expressed the experience of intense social pressure to function and to perform. These expectations had gradually changed their conception of illness and treatment to a more mechanistic perception. They used the metaphor of a mentally ill person as a “dysfunctional machine” which has to go to the “garage” where it quickly gets repaired with medication so that it can return to its “normal” tasks. Facing the system (of care/society) made them feel helpless. Young people described an inner conflict in that on the one hand they were attempting to meet social expectations, while on the other realising that a serious mental illness inevitably causes a disruption to one’s life. When confronted with older people and their situation in inpatient mental health settings this inner conflict became apparent and caused feelings of distress and despondency (“cognitive dissonance—ideal of care vs. reality”).

*Great, now I ended up here again. For me, it is a personal failure somehow. Because those who know me know that I am a person trained to achieve… (female, GD: inpatient care after the transition process).*

*You get sicker in a locked ward than you were beforehand. Because (…) you go there and you are actually doing well, you only had bad thoughts; and then some random people approach you and they are totally messed up and you think to yourself, yes, great, soon I will be like them (male, GD: outpatient care after the transition process).*

Participants revealed that they had a clear understanding of a broad conception of mental illness, accepted different aetiological models, and wanted this diversity to be represented in care provision. But in reality, participants described that they felt they often were confronted with one-sided medication treatment, especially in adult psychiatric inpatient care (“concept of mental illness”).

*To simply have more options, the feeling that one can do something to help oneself. Because I don’t know what to do when I want help (male, GD: inpatient care after the transition process).*

### Research question 2: health care needs

Participants expressed diverse demands regarding care, i.e. care should address many areas based on a broad conception of mental health problems. Care should be authentic, individualised, supportive, and intensive (“expectations of care”).

They described the need for personal closeness to the provider, to their friends as well as to daily life. The development of individualised bonds and the experience of personal support and engagement from providers was an element of the care process that participants appreciated with particular emphasis (“need for closeness”).

*Good psychologists do more for you than they have to. They appreciate you, show empathy but they also disclose their mistakes to you; they are honest with you, normal psychologists listen to you (…) bad psychologists destroy you (female, GD: currently not in treatment before the transition process).*

*You go there for one hour per week and you get there and say: yes, I’m fine today. They do not notice when someone’s not doing well because they simply do not see you in the rest of your life (female, GD: inpatient care before the transition process).*

Furthermore, participants emphasised the importance of recognising their autonomy and their right to be informed about treatment decisions and about the transfer and privacy of their personal data. Further topics addressed were the need for more information about patient rights, mental health diagnosis and treatment options (“maturity and the right to have a voice”).

### Research question 3: factors influencing health behaviour

Study participants indicated three reasons which might negatively influence their health behaviour. First, they reported stigmatising experiences (from their direct social environment, from society and from their own view about themselves) and expressed uncertainty regarding how to deal with it (“Stigma”).

*Then I am allowed to go to my family again, to celebrate Christmas; where everybody is like: Oh, it’s not that bad and don’t make such a fuss about it (male, GD: outpatient care after the transition process).*

*Well, society does not like something like that; they always want to see and hear “the normal”. What people consider normal is not what I regard as normal, yes, normal things are boring (female, GD: currently not in treatment after the transition process).*

Secondly, in their role as patients in the care system, study participants described how they are treated more like objects than individuals. In contrast, they expressed their wish to establish good relationships with care providers on an equal footing and in a care setting that treated them with respect (“objectification of patients through care providers”).

Thirdly, as a result of being disappointed by the care system, participants described their reactions in dealing with unaddressed needs or barriers and attitudes towards help-seeking as having become predominantly passive. They highlighted episodes in which they made fun of the system, in which they closed themselves up or were not telling the truth about their real inner state, in which they broke off contact with the system or sought help elsewhere, e.g. by believing in God (“strategies for coping with burden”).

*I’m not someone who doesn’t believe in medical treatment… I say that medical care comes from God, and the sciences and the intelligence of many doctors; I think all these things are a gift. But I found that for me psychotherapy is not necessary. I have tried it, but I think it is not beneficial for me now (female, single interview: currently not in treatment after the transition process)*

## Discussion

The study provides insights into the young user perspective of mental health care during the transition from child and adolescent to adult care, on health care needs and on factors influencing health behaviour.

### Perception of health care

A principal finding is that the basic attitude of the participants in our study toward professional mental health care services is that they are pessimistic and disillusioned. This is not directly in line with previous research which had a greater focus on either the practical aspects of transitioning [22, 23] or involvement in a supporting environment [20, 21]. Young people in our study felt more isolated and focussed on drawbacks and discrepancies between their perception of the care system and reality as perceived by others. Beyond that aspect, young people in our study rather focussed on themselves as independent individuals instead of on being part of a supporting environment (e.g. caregivers).

Participants expressed their wish for an attentive and open contact with professionals on equal terms. They also emphasised the importance of empathy and professional, “parental-like” support which they felt was rarely provided. These experienced shortcomings tended to leave them feeling hopeless and powerless, especially when they perceived the system as ‘superior’ and lacking any room for them to act. Moreover, the experience of failing to meet expectations of self-optimisation might have caused frustration and anger towards the mental health services system. These results indicate that integrating the mental illness into their personal identities and lives in terms of a recovery process is a permanent challenge for transition-age youth [29].

### Health care needs

Participants expressed complex and demanding health care needs for trusting and close therapeutic connections. In line with previous findings [17, 18, 30], participants strongly emphasised relationship issues by referring to numerous examples of positive and negative encounters with mental health care providers. At the same time, participants also stressed their need for interpersonal relationships with family and peers for coping with their mental illness. Previous studies have shown that factors such as feeling connected with significant others and the presence of social support affects health behaviour and protects against mental health risks [31–33]. Social support from any source (family, peers or care providers) can be of great importance to cope with stressors and daily challenges and to promote a sense of hope [32]. Mental health care providers need special therapeutic skills and training to effectively interact with young people and to help them engage with care more. Findings from this study clearly add to existing evidence indicating that professionals in AMHS particularly should be routinely provided with such training [33].

Participants called for a stronger involvement in decisions regarding their own treatment, which seems to contrast with the lack of active coping strategies paralleled by a lack of health literacy, a finding in this study and in previous research [13, 14]. This implies that young patients should be better educated about the health care system, about the transition between care systems in order to strengthen their health competence and self-efficacy [34]. Such programmes should include information about patient rights and responsibilities when coping with mental health problems, and putting across a clear picture about what AMHS are able to provide in order to avoid frustration [21]. Where needed, AMHS professionals should also provide more “prolonged parenting”. This means offering more concrete assistance to young adults, and to initiate discussion about maturation and normative developmental tasks.

### Health behaviour

Participants’ description of their health behaviour is predominantly characterised by a withdrawal from the system and with mainly passive reactions and coping strategies. One reason for that could be the disappointing experiences which they made in the health care system. The experience of being treated as an impersonal object and the experience of stigmatisation from within the mental health care system as well as from outside (families, relatives, peers, the education system, work, society at large) may account for strategies of avoidance and distancing as emphasised in previous findings [17, 19]. This might lead to young people perceiving professional health care as not beneficial and discontinuing treatment [35].

Self-determination theory is a can be drawn on to provide a better understanding of this [36, 37]. A basic lack of motivation for help-seeking from a professional mental health service in the study seems to be an expression of frustration and lack of satisfaction in terms of the three basic parameters of SDT: autonomy, relatedness and competence. If young patients experience a high system dependency, an indifferent and distant system in which they have no say in their own treatment, then they turn their backs on the system. Previous research has also found that among transition-age youth stigmatising experiences and coping are major issues accompanied by high levels of uncertainty [16, 19]. Social rejection and failing to achieve goals may result in feelings of insufficiency and loneliness. A lack of positive role models for dealing with mental health issues or overcoming crises could lead to young people generalising such experiences, and finally giving up help-seeking due to learned helplessness [7]. Perceiving mental illness as a sign of personal weakness rather than an illness may compromise active help-seeking and reduce positive beliefs about professional sources [38]. One possibility for health care professionals to prevent these negative processes might be putting more emphasis on forming stable relationships in personal encounters. As mentioned above, staff training programmes need to be developed and evaluated in order to address these important issues.

### Strengths and limitations

A main strength of the study is its open, narrative approach, which ensured that the group discussions were dynamic through self-monitoring. Implicit knowledge and orientations could thus be accessed. Furthermore, group discussions and interviews with young adults were conducted in different, multi-professional treatment settings (in a university hospital as well as in outreach and community clinics) with varying entry requirements. Hence the spectrum of (user) expert experiences recorded and analysed was wide-ranging. Experiences with care providers in multi-professional teams helped to reflect the reality of current mental health care.

The study’s limitations are the following. Because of the unique characteristics of the German health care system, the results from this small qualitative trial must be regarded as country-specific. However, similar themes have been reported in comparable studies from different care systems. We sought to reach theoretical saturation but due to research constraints this could not fully be reached. Group discussion as a qualitative method has some methodological limitations. Due to group dynamic processes, the method implies a risk for dominant personalities to override quiet voices. Some participants might be hesitant about voicing certain opinions when they do not know the other participants. In order to minimise the risk, we offered the possibility of a personal interview in some cases. Since the process of group discussions was supposed to be very self-directed, it supported a process of formulating social consensus and stereotyping which may be shaped by a certain language of authenticity which might sound rigorous and drastic. An extension of the purely qualitative approach would be a mixed-methods approach where a quantitative component would be used to verify the findings. Furthermore, caution must be exercised when drawing conclusions from the results since participants are at different stages of the transition and their individual perceptions reflect a snap-shot of the process. At last, people who did not speak German well enough were excluded but are an interesting group for further research in the field.

## Conclusions

Our findings imply that in order to alleviate the disenchantment with professional health care prevalent among transition-age youth, staff training should put more emphasis on how to form and maintain stable connections along with an open style of communication. Such constructive partnerships may constitute the basis for instilling hope and implementing trusting and joint care planning. Our findings also suggest that interventions need to be developed and evaluated to effectively strengthen young people’s individual health related competences, behaviour and self-efficacy to enable them to better cope with stigmatising experiences. Such interventions may include the involvement of persons from their more intimate social environment or peer counsellors as partners in treatment. An environment supporting autonomy in treatment with a focus on young people’s initiatives, choices and treatment decisions could increase motivation and proactive behaviour. This will help strengthen and encourage transition-age youth with mental illness in their self-reflective capacities and in their right to actively participate in their care process.

## Additional file

**Additional file 1: Figure S1.** Full coding tree as a mind map with 4 levels.

## Abbreviations

AMHS: adult mental health service
CAMHS: child and adolescent mental health service
COREQ: consolidated criteria for reporting qualitative research
SDT: self-determination theory
