# Supplementary figures and images for: Lost in transition? Perceptions of health care among young people with mental health problems in Germany: a qualitative study
Source: Child Adolesc Psychiatry Ment Health. 2018 Aug 7;12:41. doi: 10.1186/s13034-018-0249-9 (PMC6080358; doi:10.1186/s13034-018-0249-9)

Figure S1: Full coding tree as a mind-map with 4 levels


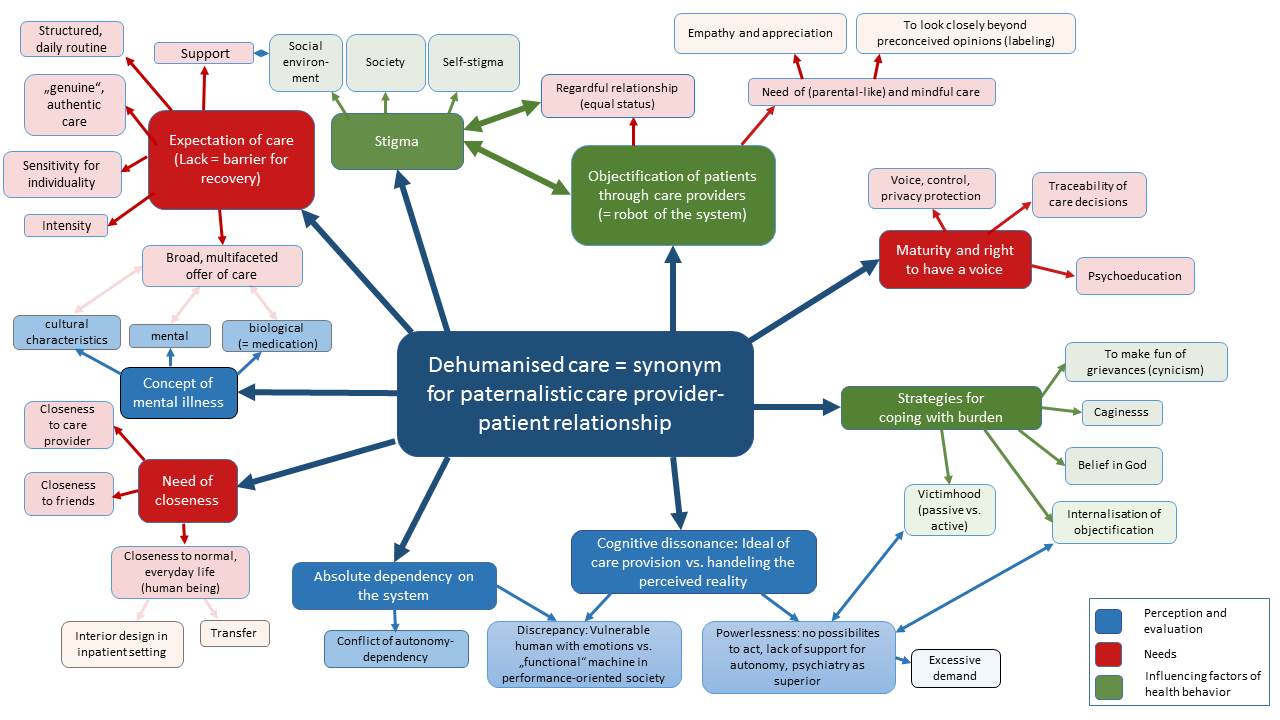

Supplement: Supplementary file 1 — Additional file 1: Figure S1. Full coding tree as a mind map with 4 levels. [file 13034_2018_249_MOESM1_ESM.docx]
